# The Effect of Type of Femoral Component Fixation on Mortality and Morbidity after Hip Hemiarthroplasty: A Systematic Review and Meta-Analysis

**DOI:** 10.1007/s11420-020-09769-1

**Published:** 2020-08-05

**Authors:** Mohamed Imam, Mohamed Shehata, Mahmoud Morsi, Muhammad Shawqi, Ahmed Elsehili, Paul Trikha, Lukas Ernstbrunner, Ashwin Unnithan, Arshad Khaleel, Puneet Monga, Ali Narvani, Asser Sallam

**Affiliations:** 1 Rowley Bristow Orthopaedic Centre, Ashford and St. Peter’s NHS Trust, Chertsey, UK; 2 University of East Anglia, Norwich, UK; 3 Faculty of Medicine, Zagazig University, Zagazig, Egypt; 4 Faculty of Medicine, Menoufia University, Menoufia, Egypt; 5 Faculty of Medicine, Assuit University, Assuit, Egypt; 6 Department of Orthopaedics, Balgrist University Hospital, University of Zurich, Forchstrasse 340, 8008 Zurich, Switzerland; 7 Upper Limb Unit, Wrightington Hospital, Wigan, UK; 8 Department of Trauma and Orthopedic Surgery, Suez Canal University Hospitals, Kilo 4.5 Ring Road, Ismailia, 41111 Egypt

**Keywords:** Hip hemiarthroplasty, Cemented, Uncemented, Mortality, Morbidity

## Abstract

**Background:**

Hip hemiarthroplasty is a well-established treatment of displaced femoral neck fracture, although debate exists over whether cemented or uncemented fixation is superior. Uncemented prostheses have typically been used in younger, healthier patients and cemented prostheses in older patients with less-stable bone. Also, earlier research has suggested that bone cement has cytotoxic effects and may trigger cardiovascular and respiratory adverse events.

**Questions/Purposes:**

The aim of this systematic review and meta-analysis was to compare morbidity and mortality rates after cemented and uncemented hemiarthroplasty for the treatment of displaced femoral neck fractures in elderly patients.

**Methods:**

Using the Preferred Reporting Items for Systematic Reviews and Meta-Analyses (PRISMA) guidelines, we searched seven medical databases for randomized clinical trials and observational studies. We compared cemented and uncemented hemiarthroplasty using the Harris Hip Score (HHS), as well as measures of postoperative pain, mortality, and complications. Data were extracted and pooled as risk ratios or standardized mean difference with their corresponding 95% confidence intervals in a meta-analysis model.

**Results:**

The meta-analysis included 34 studies (12 randomized trials and 22 observational studies), with a total of 42,411 patients. In the pooled estimate, cemented hemiarthroplasty was associated with less risk of postoperative pain than uncemented hemiarthroplasty. There were no significant differences between groups regarding HHS or rates of postoperative mortality, pulmonary embolism, cardiac arrest, myocardial infarction, acute cardiac arrhythmia, or deep venous thrombosis.

**Conclusions:**

While we found that cemented hemiarthroplasty results in less postoperative pain than uncemented hemiarthroplasty in older patients with femoral neck fracture, the lack of significant differences in functional hip scores, mortality, and complications was surprising. Further high-level research is needed.

## CME Program Description

This HSS Journal^®^ continuing medical education (CME) activity has been developed by the journal editors. After completing this activity, learners will be able to demonstrate an increase in or affirmation of their knowledge of the relevant topic. They will also be able to evaluate the appropriateness of clinical data and apply it to their practice and the provision of patient care.

## Program Format

In each edition, the HSS Journal will contain one clinically relevant article selected by the editors to be designated for CME credit.

## Accreditation

Hospital for Special Surgery is accredited by the Accreditation Council for Continuing Medical Education (ACCME) to provide CME for physicians.

Hospital for Special Surgery designates this journalbased CME activity for a maximum of 1.0 AMA PRA Category 1 Credit™. Physicians should claim only the credit commensurate with the extent of their participation in the activity.

## Disclosure Information

None of the individuals in a position to control the content of this CME activity have any relevant financial relationships to disclose.

## Completion Requirement

To complete the activity and earn credit, the participant must read the article, complete an online knowledge assessment with a score of 100%, and complete the evaluation.

## To complete the activity and earn CME credit

Go to https://bit.ly/HSSJournalCME. Please note that first-time users will need to create a free HSS eAcademy® account to access the course page.

Register for the activity, proceed through the article, and complete the post-course knowledge assessment.

Once you have completed the knowledge assessment with a score of 100%, you will be prompted to complete an evaluation and generate your certificate of CME credit.

If you have any questions, please email professionaleducation@hss.edu or phone 212-606-1057.

## Introduction

Displaced femoral neck fractures are associated with persistent hip pain, disability, and high morbidity and mortality rates, significantly affecting quality of life [4, 5, 18]. Debate continues over the selection of prosthesis to be used for hemiarthroplasty [3, 28, 40, 43, 54, 55, 56].

Historically, the use of uncemented femoral components has been indicated in younger-elderly patients with relatively good bone quality, although disadvantages include higher risks of thigh pain and periprosthetic fracture [27, 40]. Cemented femoral components are typically used in elderly patients with poor bone quality and are associated with less thigh pain and stem loosening [53], but they have been associated with higher risks of cardiac events, deep venous thrombosis (DVT), and pulmonary embolism as a result of bone cement implantation syndrome [1, 19, 38,39, 40, 65]. Various studies have reported that bone cement can have cytotoxic effects and mediate procoagulant activities, which could trigger cardiovascular and respiratory events, the main causes of death in elderly patients with reduced reserve capacity [14, 15, 19].

Consequently, we conducted a systematic review and meta-analysis to compare the rates of mortality and complications, including pulmonary embolism, cardiac arrest, myocardial infarction, acute cardiac arrhythmia, and DVT, after cemented and uncemented hemiarthroplasty used for the treatment of displaced femoral neck fractures in older patients.

## Methods

We followed the Preferred Reporting Items for Systematic Reviews and Meta-Analyses (PRISMA) statement (www.prisma-statement.org) as our guide during the preparation of this systematic review and meta-analysis. Moreover, all steps were performed in strict accordance with the *Cochrane Handbook of Systematic Reviews of Interventions* [33].

We performed electronic searches of PubMed, the Cochrane Central Register of Controlled Trials (CENTRAL), Scopus, Embase, EBSCO, Ovid, and Web of Science in May of 2017, using the following keywords: “hemiarthroplasty,” “arthroplasty,” “femoral neck fractures,” “intracapsular hip fractures,” “hip prosthesis,” “cemented,” “cementless,” and “uncemented.” We modified terms as necessary to suit each database and applied no restrictions of publication date. We also searched the US clinical trial registry (www.clinicaltrials.gov) for additional ongoing and unpublished studies and searched the reference lists in eligible studies for relevant articles not otherwise identified.

We included randomized clinical trials and observational studies that met the following inclusion criteria: the study enrolled patients over 65 years who underwent surgery for displaced femoral neck fractures, the intervention was hemiarthroplasty with a cemented or uncemented (cementless) prosthesis, and the study compared the outcomes of cemented and uncemented hemiarthroplasty.

We excluded reviews, case reports, and duplicates, as well as studies in which patients had had a previous fracture of the same hip or a pathological fracture, in which an animal model was used, or that were not in English. Eligibility screening was conducted in two steps, each by three independent reviewers: title and abstract screening for matching the inclusion criteria and full-text screening to determine eligibility for meta-analysis. Disagreements were resolved by a third reviewer.

The outcomes of interest included hip function as assessed by the Harris Hip Score (HHS) [31, 50], postoperative pain, medical outcomes (including pulmonary embolism, cardiac arrest, myocardial infarction, acute cardiac arrhythmia, and DVT), and mortality rates at 1 month, 3 months, and 1 year after surgery.

Data were extracted from the included studies by three independent researchers using Microsoft Excel. Disagreements were resolved by discussion and consensus among senior researchers. Extracted data included first author, publication year, study design, number of participants in each group, mean age, sex, type of intervention, study period, follow-up period, and outcomes of interest. For the randomized clinical trials, we used the Cochrane Collaboration’s tool for assessing the risk of bias [33]. For observational studies, we used the Newcastle–Ottawa Scale for assessing the quality of observational studies [66], and each included study was assessed according to reporting of three essential domains: selection of the study subjects; comparability of groups, in terms of demographic characteristics and important potential confounders; and ascertainment of the prespecified outcome (exposure/treatment). To assess the risk of bias across the included studies, we compared the reported outcomes between all studies to exclude selective reporting of outcomes.

### Data Analysis

We calculated risk ratios (RRs) with 95% confidence intervals (CIs) for dichotomous outcomes and standardized mean difference (SMD) with 95% CI for continuous data. Heterogeneity was assessed using the Cochran Q test, χ^2^ test for Q statistic distribution, and the I^2^ test. We performed the meta-analysis using a fixed-effect model if no significant heterogeneity was present (I^2^ < 50%; *p* > 0.1). Otherwise, we adopted the random-effect model. Egger’s test and the trim-and-fill method were used to assess the possibility of publication bias. Data analyses were performed using the R software “meta” package, version 4.9–2 (R Foundation, Vienna, Austria), for Windows. A *p* value of < 0.05 was considered statistically significant.

## Results

**Fig. 1:**
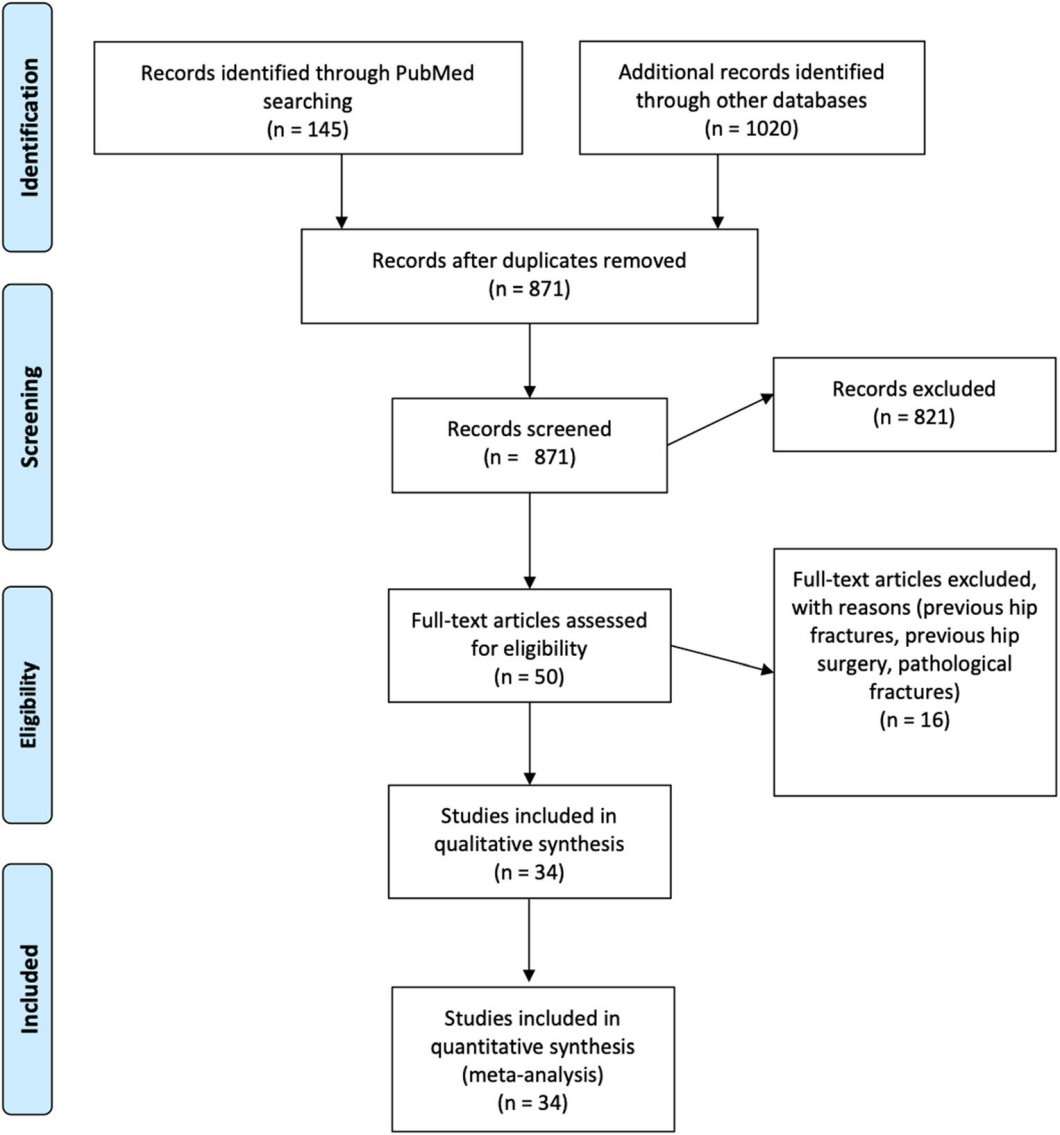
Flow diagram of study selection

The literature search yielded 871 unique records. Upon screening of titles and abstracts, 50 articles were retrieved and screened for eligibility. Of these, 34 articles were included in the meta-analysis. The study selection process is shown in the PRISMA flow diagram (Fig. 1).

**Fig. 2:**
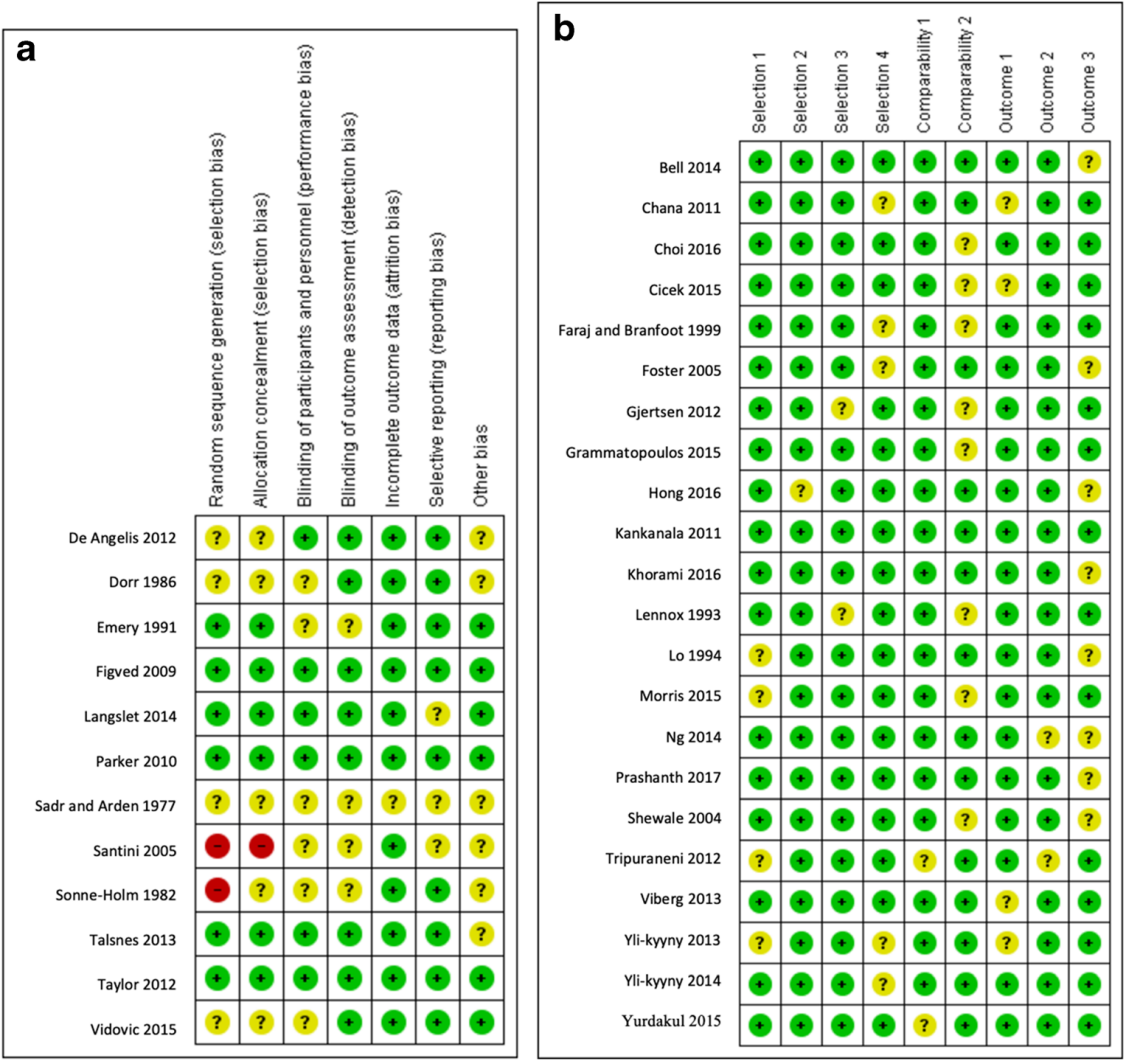
**a** Risk-of-bias summary of randomized clinical trials, according to the Cochrane Collaboration’s tool for assessing the risk of bias [33] and **b** risk-of-bias summary of observational studies, according to the Newcastle–Ottawa scale [66]

Of the 34 studies included in our analysis, 12 were randomized clinical trials [17, 20, 22, 25, 42, 55, 59, 60, 65, 68, 69, 73] and 22 were observational studies [2, 7, 9, 10, 24, 27, 29, 30, 35, 37, 40, 44, 47, 51, 52, 57, 61, 70, 72, 75–77]. The 34 included studies investigated a total of 42,411 participants; among whom 32,385 underwent cemented hemiarthroplasty and 10,026 underwent uncemented hemiarthroplasty (Table 1). The risk of bias in the randomized clinical trials was acceptable according to the Cochrane risk-of-bias assessment (Fig. 2a). The observational studies achieved a mean of 7 out of 9 points on the Newcastle–Ottawa Scale, indicating a moderate quality (Fig. 2b).

**Table 1:** Summary of included studies and selected baseline characteristics of their study population

First author, year	Study design	No. of patients (%)		Mean age, years		No. female (%)		Study period	Mean follow-up
		CH	UCH	CH	UCH	CH	UCH		
Prashanth et al. 2017 [57]	Observational study	24 (46)	28 (54)	70		30 (58)		2006–2014	59 months
Choi et al. 2016 [9]	Observational study	115 (64)	65 (36)	77	76	84 (73)	46 (71)	2009–2014	27 months
Khorami et al. 2016 [40]	Observational study	22 (43)	29 (57)	79	71.1	20 (90)	12 (41)	2011–2013	19.2 months
Hong et al. 2016 [35]	Observational study	133 (49.1)	138 (50.9)	76	75.2	104 (78.2)	100 (72.5)	2011–2013	12 months
Cicek et al. 2015 [10]	Observational study	43 (51.2)	41 (48.8)	75.65	77.52	23 (53.5)	23 (56.1)	2007–2012	46.1 months
Grammatopoulos et al. 2015 [30]	Observational study	292 (71)	120 (29)	82.1	83.4	196 (67.1)	79 (65.8)	2010–2012	12 months
Morris et al. 2015 [51]	Observational study	33 (41.25)	47 (58.75)	83.3	83.1	12 (36.4)	16 (34)	2013–2014	NA
Vidovic et al. 2015 [73]	RCT	30 (50)	30 (50)	85.39	84.97	30 (100)	30 (100)	NA	12 months
Yurdakul et al. 2015 [77]	Observational study	67 (50.4)	66 (49.6)	77.82	78.5	32 (47.8)	35 (53)	2006–2012	30.9 months
Bell et al. 2014 [2]	Observational study	110 (61.5)	69 (38.5)	82.7	82.5	45 (65.2)	45 (65.2)	2008–2010	NA
Yli-Kyyny et al. 2014 [76]	Observational study	20,682 (82)	4492 (18)	81	81	15,263 (73.8)	3315 (73.8)	1991–2009	12 months
Ng et al. 2014 [52]	Observational study	96 (46)	111 (54)	73	72	75 (78)	86 (77)	2005–2009	28.8 months
Langslet et al. 2014 [42]	RCT	112 (51)	108 (49)	83.4	83	87 (77.8)	80 (74.1)	2004–2006	60 months
Talsnes et al. 2013 [68]	RCT	162 (49)	172 (51)	84.3	84	118 (72.8)	135 (78.5)	2005–2010	12 months
Viberg et al. 2013 [72]	Observational study	209 (50.7)	203 (49.3)	83	84	169 (80.9)	163 (80.3)	1991–1995	15.5 years
Yli-Kyyny et al. 2013 [75]	Observational study	122 (55)	100 (45)	76.8	83.1	84 (68.9)	72 (72)	2005–2006	37.4 months
DeAngelis et al. 2012 [17]	RCT	66 (50.8)	64 (49.2)	81.8	82.8	52 (78.8)	48 (75)	2005–2008	12 months
Gjertsen et al. 2012 [29]	Observational study	8639 (77.7)	2477 (22.3)	83.5	83.8	6450 (74.7)	1825 (73.7)	2005–2010	19.8 months
Taylor et al. 2012 [69]	RCT	80 (50)	80 (50)	85.3	85.1	57 (71.3)	53 (66.3)	2006–2008	24 months
Tripuraneni et al. 2012 [70]	Observational study	49 (52)	45 (48)	81.6	81.7	NA		2006–2010	24 months
Chana et al. 2011 [7]	Observational study	153 (27.3)	407 (62.7)	82		459(82)		2000–2006	3 months
Kankanala et al. 2011 [37]	Observational study	30 (27.3)	80 (72.7)	79.63	83.06	22 (73)	65 (81)	2006–2006	34.8 months
Parker et al. 2010 [55]	RCT	200 (50)	200 (50)	83	83	161 (80)	147 (73)	2001–2006	60 months
Figved et al. 2009 [25]	RCT	112 (51)	108 (49)	83.4	83	87 (78)	80 (74)	2004–2006	12 months
Santini et al. 2005 [60]	RCT	53 (50)	53 (50)	82.09	79.68	40 (75.5)	42 (79.2)	2000–2001	12 months
Foster et al. 2005 [27]	Observational study	174 (71)	70 (29)	80	83	138 (79.3)	52 (74.28)	2001–2002	NA
Shewale et al. 2004 [61]	Observational study	100 (50)	100 (50)	84.3	85.4	87 (87)	90 (90)	NA	18 months
Faraj et al. 1999 [24]	Observational study	23 (21.8)	78 (77.2)	81.7	84	18 (78.3)	72 (92.3)	1995–1997	19 months
Lo et al. 1994 [47]	Observational study	189 (42)	258 (58)	75.3	72.5	38 (20)	81 (31)	1985–1990	46 months
Lennox et al. 1993 [44]	Observational study	136 (66)	71 (34)	80	82	117 (86)	59 (83)	1989–1990	19 months
Emery et al. 1991 [22]	RCT	27 (51)	26 (49)	78	79.6	24 (89)	22 (85)	NA	17 months
Dorr et al. 1986 [20]	RCT	37 (74)	13 (26)	72	66	26 (70)	9 (69)	1980–1982	36 months
Sonne-Holm et al. 1982 [65]	RCT	55 (49)	57 (51)	76		56(74.6)		1979	12 months
Sadr et al. 1977 [59]	RCT	20 (50)	20 (50)	77	78.4	13 (65)	17 (85)	NA	10 months

*CH* cemented hemiarthroplasty, *UCH* uncemented hemiarthroplasty, *NA* not available, *RCT* randomized clinical trial

**Fig. 3:**
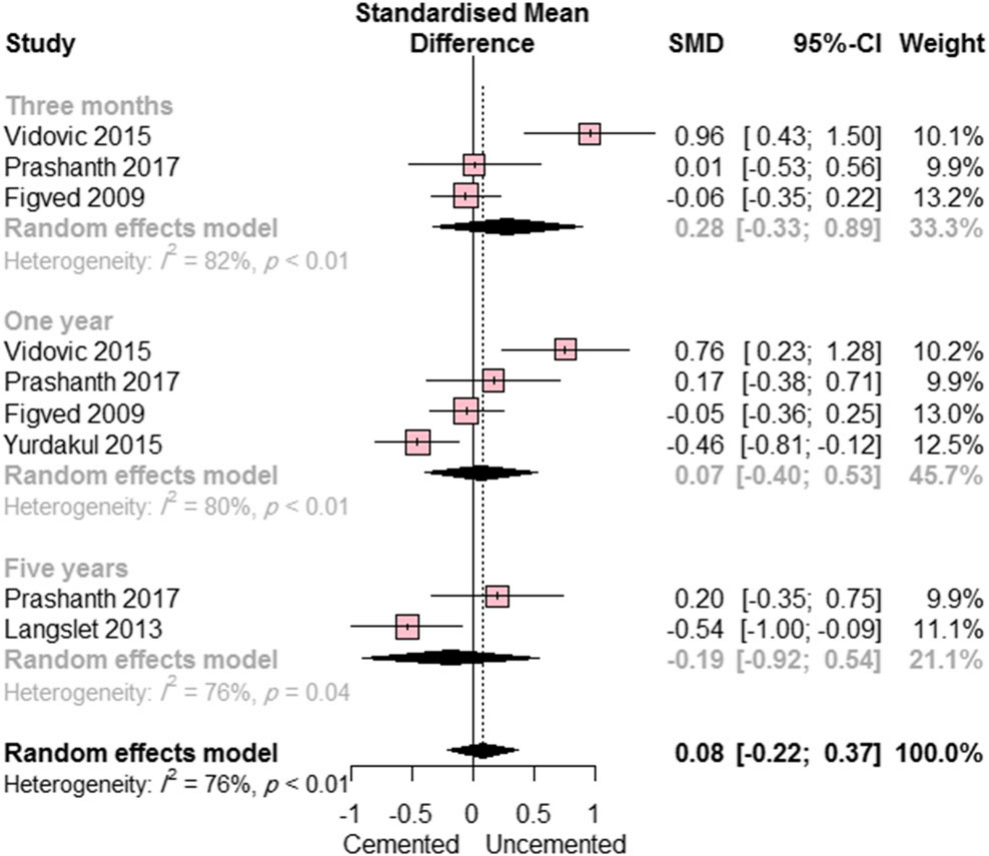
Forest plot showing the standardized mean difference (SMD) in Harris Hip Score between cemented and uncemented hemiarthroplasty (with 95% confidence interval [CI])

When the HHS was assessed (five studies: three randomized clinical trials and two observational studies), the overall estimate showed no significant difference between the cemented and uncemented hemiarthroplasty groups (SDM = 0.08; 95% CI, − 0.22, to 0.37; *p* = 0.81). This effect estimate was consistent in subgroup analyses (Fig. 3) at follow-up times of 3 months (SMD = 0.28; 95% CI, − 0.33 to 0.89; *p* = 0.23), 1 year (SMD = 0.07; 95% CI, − 0.40 to 0.53; *p* = 0.66), and 5 years (SMD = − 0.19; 95% CI, − 0.92 to 0.54; *p* = 0.17).

**Fig. 4:**
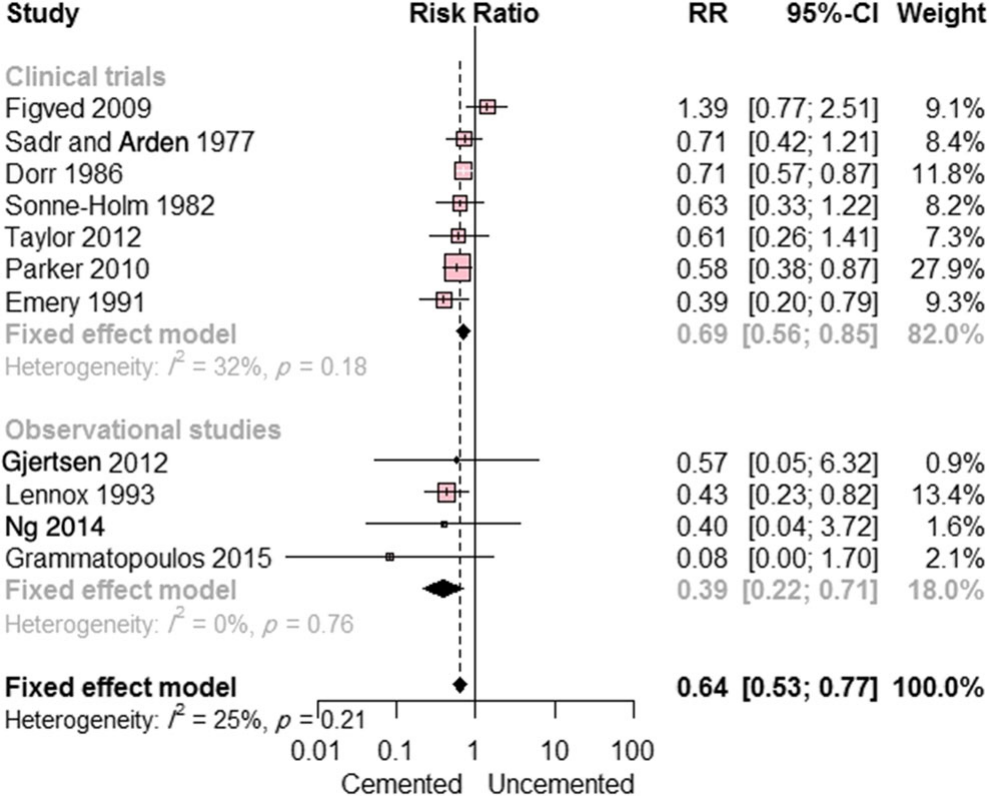
Forest plot showing the risk ratio (RR) of postoperative pain between cemented and uncemented hemiarthroplasty (with 95% confidence interval [CI])

The cemented hemiarthroplasty group was found to have a lower risk of postoperative pain. Eleven studies (seven randomized clinical trials and four observational studies) reported on postoperative pain (overall RR = 0.64; CI, 0.53 to 0.77; *p* < 0.0001). This effect estimate remained consistent in subgroup analyses according to study design (Fig. 4); no significant heterogeneity was observed (I^2^ = 25%; *p* = 0.21).

No significant differences in mortality were found between the cemented and uncemented hemiarthroplasty groups at any duration of follow-up.

**Fig. 5:**
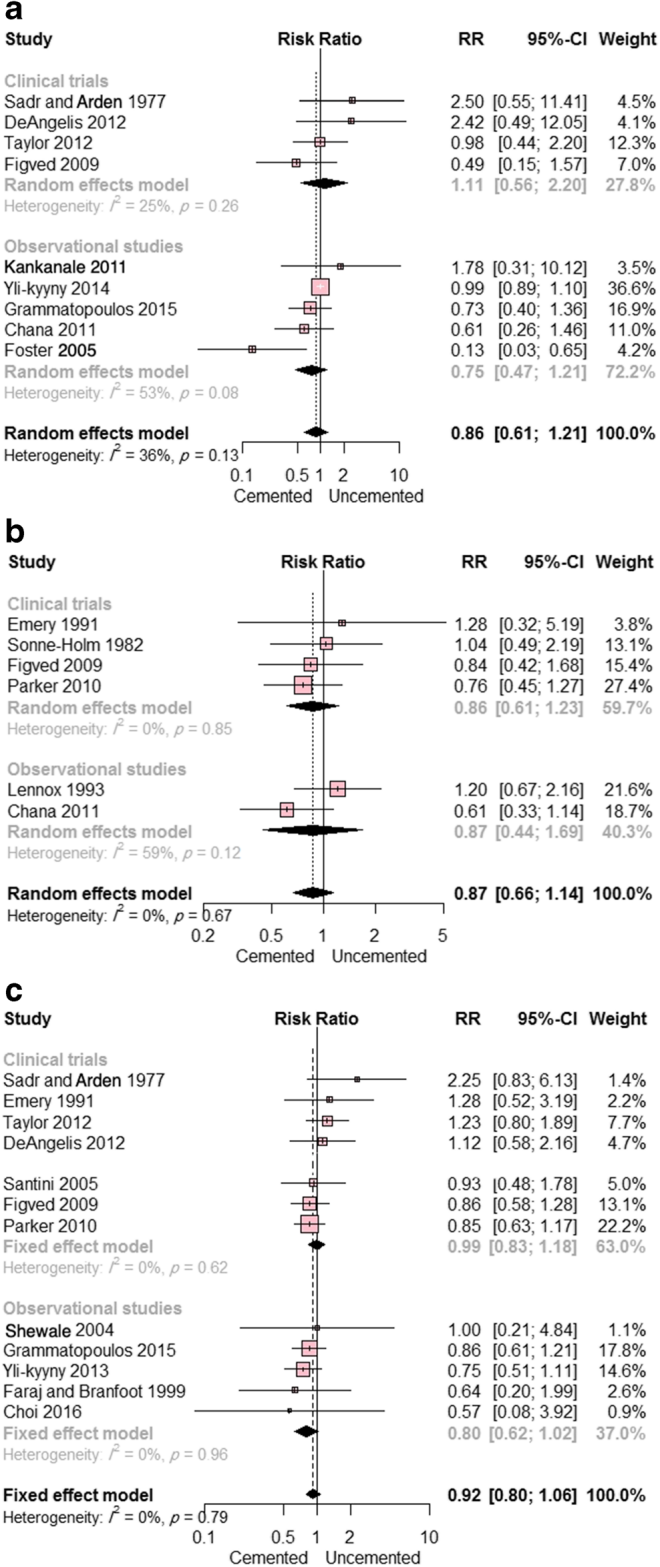
**a** Forest plot showing the risk ratio (RR) of mortality at 1 month postoperatively between cemented and uncemented hemiarthroplasty (with 95% confidence interval [CI]); **b** forest plot showing the RR of mortality at 3 months postoperatively between cemented and uncemented hemiarthroplasty (with 95% CI); **c** forest plot showing the RR of mortality at 1 year postoperatively between cemented and uncemented hemiarthroplasty (with 95% CI)

Nine studies (four randomized clinical trials and five observational studies) reported on mortality at 1 month postoperatively. There was no significant difference between the two groups (RR = 0.86; 95% CI, 0.61 to 1.21; *p* = 0.39); there was moderate heterogeneity (I^2^ = 36%; *p* = 0.32). This result remained consistent in subgroup analysis according to study design (Fig. 5a).

Six studies (four randomized clinical trials and two observational studies) reported on mortality at 3 months postoperatively. The overall pooled RR did not favor either of the two groups (RR = 0.87; 95% CI, 0.66 to 1.14; *p* = 0.31); there was no evidence of heterogeneity (I^2^ = 0%; *p* = 0.69). This result remained consistent in subgroup analysis according to study design (Fig. 5b).

Data on mortality at 1 year postoperatively were reported in 13 studies (eight randomized clinical trials and five observational studies), with no significant difference between cemented and uncemented hemiarthroplasty reported (RR = 0.92; 95% CI, 0.80 to 1.06; *p* = 0.25) and no evidence of heterogeneity (I^2^ = 0%; *p* = 0.82). This result remained consistent in subgroup analysis according to study design (Fig. 5c). Egger’s test showed no evidence of publication bias; *p* = 0.31.

**Fig. 6:**
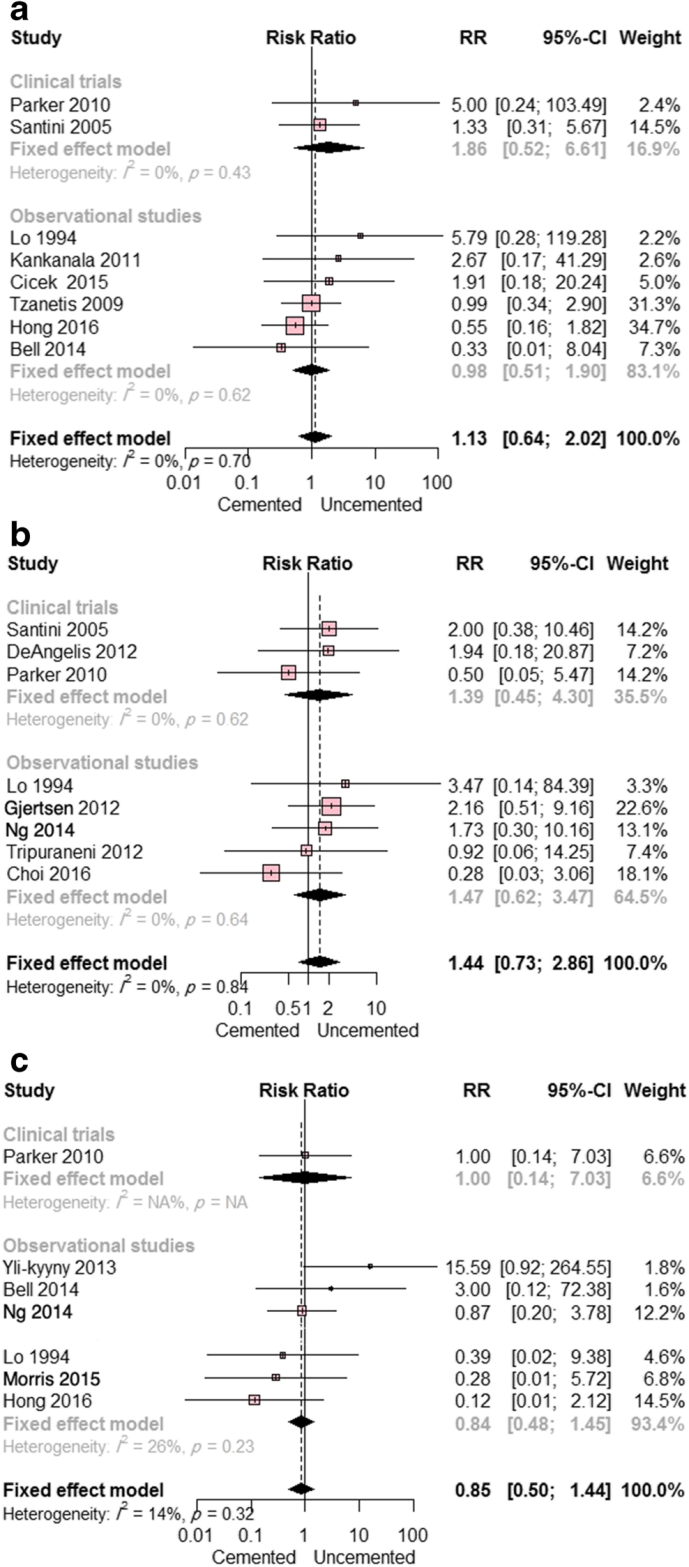
**a** Forest plot showing the risk ratio (RR) of pulmonary embolism between cemented and uncemented hemiarthroplasty (with 95% confidence interval [CI]); **b** forest plot showing the RR of myocardial infarction between cemented and uncemented hemiarthroplasty (with 95% CI); **c** forest plot showing the RR of deep venous thrombosis between cemented and uncemented hemiarthroplasty (with 95% CI)

No difference in the rates of pulmonary embolism or DVT was found. Eight studies (two randomized clinical trials and six observational studies) reported data on pulmonary embolism. The overall RR did not favor either prosthesis type (RR = 1.13; 95% CI, 0.64 to 2.02; *p* = 0.67). This result remained true regardless of study design. Pooled studies were homogeneous (I^2^ = 0%; *p* = 0.70) (Fig. 6a). Eight studies reported data on DVT (one randomized clinical trial and seven observational studies). The overall RR did not differ significantly between the two groups (RR = 0.85; 95% CI, 0.50 to 1.44; *p* =0.54); there was no notable heterogeneity among these studies (I^2^ = 14%; *p* = 0.32) (Fig. 6c).

No difference in the risk of cardiac complications was found between the two groups. Two studies (one randomized clinical trial and one observational study) reported on cardiac arrest. The combined RR did not favor either of the two groups (RR = 1.74; 95% CI, 0.13 to 23.19; *p* = 0.68). Pooled studies were heterogeneous (I^2^ = 60%; *p* = 0.67). Similarly, the overall RR in the eight studies reporting data on myocardial infarction (three randomized clinical trials and five observational studies) was comparable between the two groups (RR = 1.44; 95% CI, 0.73 to 2.86; *p* = 0.30). This result remained consistent in subgroup analysis according to study design. The eight pooled studies were homogeneous (I^2^ = 0%; *p* = 0.84) (Fig. 6b). Two studies (one randomized clinical trial and one observational study) provided data on acute cardiac arrhythmia. The combined RR did not favor either cemented or uncemented hemiarthroplasty (RR = 0.57; 95% CI, 0.08 to 4.33; *p* = 0.59). This effect estimate remained consistent in subgroup analysis according to study design. No heterogeneity was observed (I^2^ = 0%; *p* = 0.64).

## Discussion

This study of 42,411 older adults showed no significant differences between cemented and uncemented hemiarthroplasty of the hip in terms of HHS, mortality, or medical complications. However, the results did reveal cemented fixation to be associated with less postoperative pain than uncemented fixation.

Similar to our results, those of a study from the Swedish Hip Arthroplasty Register by Rogmark et al. showed no significant difference in mortality according to femoral fixation type at 1 year after surgery [58]. In addition, two recent meta-analyses, one with five randomized clinical trials [71] and the other with seven [46], reported no significant differences in mortality related to type of fixation (cemented or uncemented) 1 year after surgery. In contrast, a study of data from an Australian registry reported higher mortality on the first postoperative day in patients with cemented prostheses but an overall lower rate of death through 1 year of follow-up [12].

Several studies have described comorbidity, older age, male sex, delayed surgery, and cognitive impairment as some of the most important risk factors for death after hip fracture [34, 36, 56, 62,63, 67,68]. Our patient population may lack the reserve capacity that is essential to handle the double trauma of a hip fracture and subsequent surgery. Therefore, the more severe the comorbidity, the higher the risk of a fatal outcome when cementation is applied; these factors, of course, influence the selection of the method of fixation [68]. Nevertheless, recent improvements in surgical techniques, the careful elimination of any cellular debris and blood clots from the medullary canal before inserting the bone cement, perioperative monitoring of patients by an experienced anesthesia team, and thromboprophylaxis may well have improved the survival of hip surgery patients [21, 26, 32, 43, 64, 69] and help explain our results regarding mortality at 1 month, 3 months, and 1 year after surgery.

Earlier research has suggested that cemented femoral components in hip replacement surgery in patients with femoral neck fractures are associated with cardiovascular complications, including embolism and arrhythmia [22, 23, 45, 48, 65]. Nevertheless, our study found no differences between cemented and uncemented hemiarthroplasty in rates of postoperative myocardial infarction, acute arrhythmia, cardiac arrest, or pulmonary embolism. Our findings are supported by meta-analyses conducted by Li et al., Lin et al., and Luo et al., which found no differences in major postoperative complications between patients with cemented and cementless stems [46, 47, 49].

High risks of DVT after cemented hip and knee arthroplasty have been reported in earlier studies [13, 41, 49]. The hypercoagulable state that follows femoral neck fracture is associated with an increased risk of thromboembolism, and factors enhancing hypercoagulability include, in addition to the initial trauma, subsequent surgery, blood loss resulting from either fracture or surgery, and perioperative fluid therapy [74]. Furthermore, the thrombogenic properties of the bone cement contribute to the pathogenesis of DVT. Polymethylmethacrylate monomer found in mixed venous blood during cemented arthroplasty induces secretion of platelet activation factors, such as transforming growth factor β and β-thromboglobulin, and stimulates monocytes to express tissue factor, which triggers coagulation [6, 16]. Additionally, higher levels of cytokine CD14/42a, a known measure of platelet–monocyte aggregation, are present in patients undergoing cemented arthroplasty [8]. In contrast, a small study by Hong et al. reported no statistically significant difference in DVT development between cemented and uncemented hemiarthroplasty prostheses used to treat traumatic displaced femoral neck fractures (3.0% [*n* = 4] and 5.1% [*n* = 7], respectively) [35].

Clarke et al. studied the bone cement as a risk factor for DVT, comparing three sets of patients undergoing a cemented or uncemented total knee replacement (TKR) or a cemented total hip replacement (THR) [11]. They found that uncemented prostheses were associated with a greater prevalence of DVT at 5 to 7 days than cemented prostheses, and both knee groups had a significantly higher prevalence of DVT than the cemented THR group. The thrombi were significantly longer after cemented TKR (26.5 cm) than after both uncemented TKR (11 cm) and cemented THR (7 cm). The authors concluded that the bone cement may influence the length of a thrombus but does not lead to an increase in the incidence of DVT.

Some study limitations exist. It is possible that unbalanced cohort sizes and the use of different types of hip prosthesis limit the study’s power to detect differences between cohorts. Additionally, comorbidity as a risk factor for death was not well assessed in all of the included studies. Another limitation is the inclusion of only English-language literature; relevant studies in other languages might have been omitted. Finally, causality cannot be determined in observational studies, limiting the conclusions of our meta-analysis.

In conclusion, current evidence shows patients treated with cemented hemiarthroplasty experience less postoperative pain than those treated with uncemented prosthesis. Our meta-analysis shows no significant differences between cemented and uncemented hip hemiarthroplasty in terms of functional hip score (the HHS); postoperative mortality; or medical complications, including pulmonary embolism, cardiac arrest, myocardial infarction, acute cardiac arrhythmia, and DVT. The absence of a connection between cemented prostheses and complications is surprising, considering earlier research findings and the use of cemented femoral components historically in older patients with poor bone quality and greater comorbidity. As surgical techniques and perioperative care continue to improve, further research should be conducted to confirm our findings.
